# Hydration Strategies in Older Adults

**DOI:** 10.3390/nu17142256

**Published:** 2025-07-08

**Authors:** Jacquelyn Pence, Allyson Davis, Ebonie Allen-Gregory, Richard J. Bloomer

**Affiliations:** Center of Nutraceutical and Dietary Supplement Research, College of Health Sciences, University of Memphis, Memphis, TN 38152, USA; jpence1@memphis.edu (J.P.); laboswll@memphis.edu (A.D.); eallen10@memphis.edu (E.A.-G.)

**Keywords:** dehydration, hydration, older adults

## Abstract

Hydration is the body’s ability to absorb water and to maintain the correct balance of fluid and electrolytes and is essential to human health. Dehydration can adversely affect metabolism, thermoregulation, digestion, and neurological, kidney, and heart function. Aging as well as disease and medications affect water and electrolyte levels in the body and can lead to dehydration in older adults. In this review, we discuss factors contributing to dehydration in older adults, how hydration is measured, and strategies to improve hydration status. We close with a summary of the different areas of focus related to hydration research.

## 1. Introduction

Dehydration is prevalent among adults aged 65 years and older. Individuals over the age of 50 are significantly less likely to comply with adequate fluid intake than their younger counterparts [1], and 25–33% of adults in the United States and Europe consume less than 1.5 L of fluid per day [2]. Large-scale surveys consistently indicate that a significant proportion of older adults (aged 65 and over) do not meet recommended fluid intake levels. In a multi-country analysis involving 16,276 participants—7580 men and 8696 women—from 13 countries, approximately 50% reported inadequate fluid intake, with older adults particularly affected. The French population was identified as being at higher risk, especially during periods of extreme summer heat, highlighting concerns about suboptimal hydration [1]. Similarly, data from the National Health and Nutrition Examination Survey (NHANES) indicate that habitually low fluid consumption is common among community-dwelling older adults. Furthermore, both thirst perception and fluid intake tend to decline with age [3]. Armstrong et al. also emphasize that inadequate hydration is widespread and becomes increasingly problematic with advancing age [2].

While reported dehydration rates vary widely, likely due to the use of different criteria to assess dehydration, a 2023 meta-analysis found that nearly one in four older adults were dehydrated and that these rates increased to one in three for individuals in long-term care or with pre-existing illnesses [4]. Similarly, while 0.5–1.5% of older adults are admitted to the hospital specifically for dehydration [5,6] (one of the top reasons for older adult hospital admission), a third of older adults have been found to be dehydrated [7]. While there has not been a recent published evaluation of dehydration-related healthcare costs, it has previously been estimated to raise hospitalization costs by 7–8.5%, and associated hospitalization costs were estimated to be over USD 1 billion per year within the United States [8,9]. Dehydration during hospitalization has been linked to higher morbidity and mortality [6], longer hospital stays [8], and increased hospital-associated disability from baseline to 3 months post-discharge [10]. Older adults presenting with pneumonia and dehydration were two times more likely to die within 6–12 months following pneumonia [11].

### 1.1. What Is Hydration, and How Does It Change with Aging?

Simply put, hydration is the body’s ability to absorb water and to maintain the correct balance of fluid and electrolytes. The content of body water is often cited as approximately 60% [5,12]. However, it has been shown to decrease with age due to muscle mass loss. One study found that water constituted approximately 62% and 55% of total body weight for males and females, respectively, during much of adulthood and decreased after 60 years of age by nearly 5% [13]. Euhydration can be defined as body water at or near equilibrium, with similar amounts of water being taken into or generated by the body as it is lost. This is sometimes defined as above a specific percentage loss of body mass, such as greater than 2% [14]. Water plays an active role in human physiology—acting as a solvent, a carrier/transporter of nutrients and waste products, and as an essential contributor to thermoregulation [12,15]. Hydration in older adults promotes mobility by reducing the risk of falls and improving orthopedic rehabilitation outcomes [16]. It helps maintain muscle function and lowers the risk of exhaustion, urinary infections, and gastrointestinal complications [17]. Additionally, dehydration can worsen conditions like hyponatremia, which is linked to cognitive impairment, exhaustion, and increased risk of falls [18].

**Figure 1:**
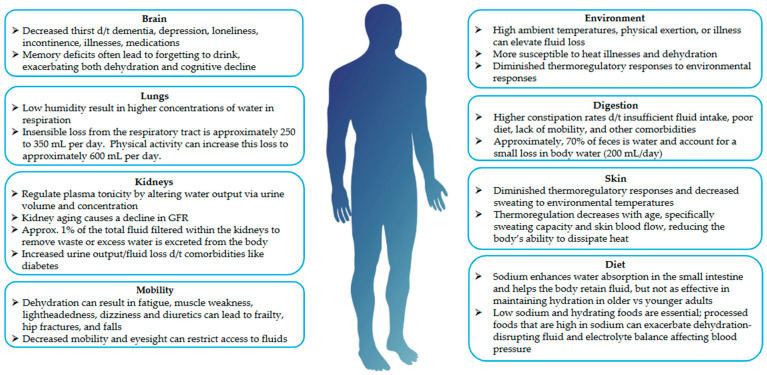
Fluid volume variables and the effects of aging. References: Brain [8,12,21,22]; Lungs [22,23,24,25]; Kidneys [26,27,28,29]; Mobility [8,12,15,16,30,31]; Environment [7,32,33]; Digestion [12,28]; Skin [33,34,35,36]; Diet [37,38,39,40,41].

Dehydration is a net loss of normal body water. The degree of hydration is sometimes further distinguished by physiological processes and biomarkers. For example, underhydration is when physiological processes are actively maintaining water and electrolyte balance in normal ranges while body water is in a deficit, and dehydration is when the deficit of body water is significant enough that normal ranges are no longer maintained [19]. These defined biological ranges of hydration biomarkers vary within research [20]. Plasma osmolality ranges are <275 mOsm/kg (overhydration/hyperhydration), ~275–295 mOsm/kg (euhydration), 295–300 mOsm/kg (underhydration), and ≥290 mOsm/kg or >300 mOsm/kg (dehydration/hypohydration). Instances of dehydration can further be categorized by coinciding changes in electrolyte levels: hypotonic, isotonic, and hypertonic. Insufficient (low) fluid intake as well as excessive sweating can lead to hypertonic dehydration, which occurs when the body loses both water and electrolytes but lesser amounts of electrolytes. Other causes of dehydration are from acute and chronic illnesses and diseases such as diarrhea, vomiting, and diabetes, as well as from medication use such as diuretics and laxatives. Causes of dehydration are discussed in greater detail below. Additionally, as seen in Figure 1, aging can alter hydration status, resulting in negative health outcomes.

Water is predominantly introduced into the body via absorption from the diet across the mucosal barrier in the small intestine, with lesser amounts in the colon [28]. Fluid intake accounts for approximately 1.5–3 L of water per day [12,42]. Food also provides approximately 0.5–1 L of water from the water content; however, the water content of solid foods is not as readily absorbed in the small intestine as fluids [43]. Macronutrient hydrolysis and oxidative metabolism generate additional water. For every 100 g of protein, carbohydrate, or fat consumed, approximately 41, 55, or 107 g of water, respectively, are generated, accounting for 250–350 mL water taken in [12,44]. Thus, the total daily water inputs for an adult are estimated to be in the range of 2–3 L [12] or 3–4 L [33].

The rate of water absorption within the digestive tract changes with the speed of digestion depending on nutrients within the food and beverages consumed, gastric volume, and energy density. When water is ingested alone, it can start being absorbed in as quickly as 5 min and is mostly absorbed within 20 min [45]. Different beverages can have different transit times. Half-emptying rates of liquids in the stomach have been found to range from 10 min to 1 h, while solid foods take longer, such as from 50 to 115 min depending on the food [46]. It is unclear how gastric emptying changes with aging or what effect this has on water absorption. Some research indicates that the stomach may empty faster in older adults for liquids, while other studies found increased times for both solids and liquids, and yet others found no differences at all [46]. Frailty may alter the rate of gastric emptying and further explain differences between study findings.

In addition to water from nutritional consumption, to aid digestion, the gastrointestinal tract also secretes roughly 7.5 L of fluid daily, which is also reabsorbed. In a day, around 6.5 L of water is absorbed in the small intestine, mainly in the jejunum and ileum, under osmotic and hydrostatic gradients [28]. Water absorption has many possible routes from inside the lumen, including aquaporins, carrier-mediated transporter systems, and tight junctions of enterocytes [43]. Changes in intestinal transit are also affected by osmolality and electrolyte content, mainly sodium. No change in transit rate has been detected with aging, and it is currently unclear whether physiological changes alter water processes within the small intestine [46].

In adults, 1–2 L of water is also reclaimed in the colon, likely hypertonically [28]. Approximately 70% of feces is water and accounts for a small loss in body water (200 mL daily) [12,28]. Like gastric emptying, there are contradictory findings on whether colonic transit time changes or slows with aging, although there is a mindset that inactivity or immobility may cause slowing [46]. Constipation rates are higher among the older adult population, but are attributed to insufficient fluid intake, poor diet, lack of mobility, and other health-related causes rather than digestive changes.

Once absorbed, water is distributed throughout the body, with approximately a third in extracellular fluid (three-quarters of that in interstitial fluid and the rest in plasma) and the remainder in intracellular fluid [5,12]. To monitor body water levels, osmoreceptors within the central nervous system sense plasma tonicity, the availability of particles such as sodium, potassium, and, to a lesser degree, glucose that aid in water transport across cellular membranes, resulting in an osmotic effect [26]. Kidneys regulate plasma tonicity by altering water output via urine volume and concentration [26]. Baroreceptors respond to increased tonicity by secreting vasopressin from the posterior pituitary, which increases water permeability in the kidneys, leading to lower urine output and high water recovery [47,48].

If a large bolus of water is rapidly consumed, the kidneys may clear the excessive fluid rapidly, generating dilute urine in order to protect the body [49]. Approximately 1% of the total fluid filtered within the kidneys to remove waste or excess water is excreted from the body. The daily glomerular filtration rate (GFR) is approximately 180 L [28], whereas water losses in urine excretion only total around 1–2 L [12].

In older adults, increases in vasopressin due to osmolality have been observed, and changes in circadian levels of vasopressin may be responsible for increases in nocturia [50]. Aging within the kidney causes a decline in GFR [27]. However, some research suggests that GFR function can be restored with increased fluid intake [42]. Aging also causes a decline in the kidneys’ ability to concentrate urine.

While urine accounts for the largest portion of water loss, body water is also decreased via sweating, respiration, and other secretions such as tears and sputum [42]. A murine study concluded that insensible losses may increase with aging, as overall hydration (indicated by stable body weight) remained the same between groups, while the older group had less urine and feces [24]. Insensible water loss from the respiratory tract in an unstressed adult is approximately 250–350 mL per day, though this amount can vary. However, physical activity can increase this loss to approximately 600 mL per day [22].

### 1.2. Causes and Concerns for Dehydration in Older Adults

A major concern for dehydration in older adults is low fluid intake. One commonly noted cause of low fluid intake with aging is a decrease in thirst [8,12,50]. Thirst is caused by a drop in osmotic pressure. Older adults may have a higher osmotic threshold that decreases their thirst sensation. The main recommendation is to increase fluid consumption [46], regardless of thirst. Other causes of low fluid intake include mobility and eyesight (which can restrict access to fluids), cognitive changes such as dementia, depression and loneliness, fear of incontinence, illnesses/diseases, and medication use [8,12].

### 1.3. Why Is Hydration Important?

Hydration is essential for health in individuals of all ages. Dehydration can diminish health through degeneration and decrease physiological function. Euhydration/dehydration can affect metabolism, neurological function, thermoregulation, digestion, physical/athletic performance, kidney and heart function, etc. [33,42]. Chronic dehydration is associated with adverse health effects. For example, in longitudinal studies ranging in length of 6.5–19.6 years, diabetes mellitus, CKD, and cardiovascular issues trend linearly with baseline copeptin levels whereas elevated baseline serum sodium (>142 or >144 mmol/L) is associated with CKD and other chronic diseases, heart failure, and all-cause mortality ~4–25 years post baseline [19]. In older adults, dehydration leads to poorer hospitalization outcomes and is associated with many adverse health outcomes including both physiological (e.g., skin hydration, kidney stones, constipation, fatigue) and psychological (e.g., mood, alertness, cognition) conditions [14]. Anger, hostility, confusion, depression, and tension were all found to increase with dehydration of greater than 1%, and short-term memory was affected after 2% of body mass was lost. Additionally, visual perceptual ability decreased with severe hypohydration [14].

Hydration also plays a key role in physical performance and muscle function. During exercise, staying well-hydrated helps preserve the integrity of the muscle and reduces the need for glycogen as a fuel source. Consuming carbohydrates alongside fluids can further enhance performance, offering benefits that are both independent of and complementary to hydration status [51,52,53].

Hydration is of significance to thermoregulation. Sweating is the main mechanism the body utilizes to correct heightened core temperature caused by physical activity or warm environments, which results in loss of body water and, to a lesser degree, electrolytes, leading to hypertonic dehydration [12]. Older adults can have diminished thermoregulatory responses to environmental temperatures [33], as well as decreased sweating [34].

Euhydration is also important in maintaining mobility with age. A decrease in intracellular water due to dehydration may lead to dynapenia, sarcopenia, and damage to cells, resulting in frailty [15]. Dehydration can result in fatigue, muscle weakness, lightheadedness, and dizziness, which can lead to falls [30], and falls in older adults often lead to fractures. Both dehydration and loop diuretics were shown to be positively associated with fall risk [31]. Changes in hydration as well as electrolyte levels can also lead to gait instability [15]. The prevalence of dehydration in older adults hospitalized for hip fractures in one study was 40–50%, and dehydration was associated with frailty [16].

Even minimal fluid loss can negatively impact cognitive performance, emphasizing the need for adequate hydration [25,54]. Ensuring adequate fluid intake can prevent cognitive decline. While being aware that cognitively impaired individuals may have unique hydration needs, addressing these needs through targeted hydration strategies can enhance cognitive performance and overall wellbeing.

## 2. What Controls Hydration?

### 2.1. Fluid

Water is not the sole fluid contributor to hydration. Contrary to popular belief, all fluids can affect hydration levels. This includes juices, coffee, milk, electrolyte beverages, and sports drinks [55]. Daily fluid requirement varies based on individual factors such as body size, activity level, weather, medical conditions, and medications. This makes the generalized recommendation of 64 ounces a day not a one-size-fits-all approach.

Thirst is the primary indicator for fluid intake. As an individual loses fluid, blood volume decreases, increasing the ratio of salt and other minerals in the blood [55]. Blood osmolality increases, and the brain releases an antidiuretic hormone, which prompts the kidneys to retain fluid [55].

### 2.2. Food

The water content of food contributes to daily fluid intake as well. This can include foods such as soup, fruit, vegetables, yogurt, etc. Caloric needs decrease with age, but studies suggest that inadequate nutritional intake is associated with dehydration [56]. Risk factors for malnutrition can include dysphagia, or difficulty swallowing, dementia [42], delayed gastric emptying due to altered muscle tone, and chronic inflammation [57]. It has been estimated that 70–80% of water intake comes from liquids and the remaining 20–30% from solid food [12,29].

### 2.3. Medications

Age-related medications can lead to a greater risk of dehydration. Loop diuretics inhibit sodium and chloride reabsorption in the loop of Henle, resulting in significant diuresis [58,59]. They have a strong diuretic effect that can lead to rapid fluid loss that further increases the risk of dehydration, especially in older adults with compromised renal function. Also, loop diuretics are associated with a higher risk of heat-related hospitalization along with ACE inhibitors, anticholinergics, and antipsychotics [60,61]. Thiazide diuretics act on the distal convoluted tubule to inhibit sodium reabsorption by excretion followed by water [59]. These diuretics can cause moderate fluid loss and may lead to electrolyte imbalances such as hypokalemia and hyponatremia. Potassium-sparing diuretics are less likely to cause dehydration compared to others, as they bind receptors at the aldosterone-dependent sodium potassium exchange site in the distal convoluted tubule of the nephron to inhibit sodium reabsorption without depleting potassium. While loop diuretics are potent and effective, they carry a higher risk of dehydration in older adults due to their strong diuretic effects. Thiazide diuretics also pose risks of electrolyte imbalance. Potassium sparing is generally safer in regard to hydration status in older adults.

Other medications include cardiac medications such as beta blockers, ACE inhibitors, angiotensin II receptor blockers, calcium channel blockers, and antiarrhythmics. Additionally, other classifications include NSAIDs and diabetic medications [29,59]. The pathways to dehydration in these medications can be diarrhea, increase in urine volume, decrease in thirst sensation, central thermoregulation affectation, increase in sweat production, or decrease in appetite [32,62].

### 2.4. Acute and Chronic Illness

Dehydration in older adults can occur in an acute manner due to changing weather conditions, whether it be from heat stress during warmer weather or illnesses such as influenza that come along with colder weather [63]. Influenza can cause diarrhea, high fever, increased sweating, and vomiting, all of which can deplete the body of electrolytes. Acute dehydration can have an iatrogenic cause, such as infections acquired while hospitalized, drug reactions, difficulties after surgery, or misdiagnosis [63]. Dehydration can also be a result of high levels of creatinine, which can cause acute kidney injury [29].

Chronic illness puts older adults, an already vulnerable population, at even greater risk of dehydration [42]. These risks continue to increase when an individual requires long-term care or hospitalization. Dehydration and chronic conditions such as diabetes, renal disease, dementia, and cardiac conditions can have a mutually deteriorating relationship [29]. Table 1 summarizes how different comorbidities can contribute to dehydration status.

Diabetes can cause an increase in thirst and urine output. This is due to high glucose levels. When glucose spills over into the urine, it draws more water into the kidney, leading to more urine output [83]. If this is not managed properly, dehydration can occur.

Kidney function declines with age, and some individuals with chronic kidney disease will develop polyuria, or an increase in urine output [80]. This is due to the impaired ability of the kidneys to properly rid the body of waste and fluid, resulting in compensatory methods and excess fluid loss.

Older adults with cognitive impairments, such as dementia, are particularly vulnerable to dehydration. Dementia can lead to dehydration in several ways. Impaired memory, or not having proper care, can lead to forgetfulness. Memory deficits often lead to forgetting to drink, exacerbating both dehydration and cognitive decline. Dementia can also cause damage to the part of the brain that controls hunger and thirst, leaving the individual with an impaired ability to detect when they need to eat and drink [21]. Dysphagia, or difficulty swallowing, can also be a result of dementia. This can cause the individual to develop fear around eating and drinking. Cardiac conditions can lead to fluid buildup due to the heart’s inability to pump blood properly. When this occurs, fluids may be restricted, and while that may help with the fluid buildup, it can also lead to dehydration and other complications of the heart’s electrical system [67].

### 2.5. Alteration to Thermoregulation with Age

Thermoregulation is the body’s ability to regulate core body temperature by balancing heat produced with heat loss [84]. Certain elements of thermoregulation decrease with age, specifically sweating capacity and skin blood flow, reducing the body’s ability to dissipate heat [35,36]. Additionally, older adults appear to lack the ability to correctly perceive increased physiological strain during heat exposure [35]. Adults over the age of 50 store 1.3 to 1.8 times more body heat when exposed to the same heat load as younger adults (19–30 years) [35]. This can occur during both exercise and passive heat exposure.

Additionally, comorbid diseases, and specifically heart failure and chronic lung disease, can compound the altered condition of thermoregulation in older adults [36].

### 2.6. Environment and Weather

Seasons of warm weather and occurrences of heat waves bring about a substantial increase in the number of dehydrated older adults [63]. This is due to their decreased ability to perceive how the warmer weather is affecting them, but also a diminished amount of support due to caregivers scheduling out-of-town trips and care facilities being short-staffed [63]. Heat waves are defined as prolonged periods (3 days or more) of exceedingly hot weather, or temperatures significantly higher than average for the region and time of year [85]. Higher levels of humidity, intense sunlight, and limited cloud cover add to the danger of heat waves [85].

Less considered are the effects of cold weather. A South Korean study found that older adults were dehydrated in both hot and cold months [86]. The researchers suggested that increased urine output, as well as a decrease in peripheral circulation, could cause dehydration in cold weather [23].

### 2.7. Sweating and Activity

As stated earlier, thermoregulation is the body’s ability to maintain a safe body temperature (37 ± 0.5 °C or 98.6 ± 0.98 °F). When body temperature varies, the thermoreceptors are activated in the skin, and the hypothalamic thermoregulatory center signals heat regulation mechanisms to increase or decrease body temperature to return it to baseline [84]. These regulation mechanisms include the sweat glands, blood vessels in the skin, adrenal and thyroid hormones, and behavioral changes such as reducing movement, appetite, and removing clothing. This all leads to increased heat loss and decreased body temperature [84].

Aside from altered thermoregulation as we age, there are disorders that can increase the risk of cold- or heat-related illnesses, and these can include spinal cord injuries, central nervous system disorders, and endocrine disorders [84]. Additionally, conditions such as hypohidrosis and anhidrosis cause decreased or no sweating and lead to an increased risk of overheating [87].

## 3. How Is Hydration Measured?

### 3.1. Crude Measures

Measuring hydration levels outside of a clinical or research setting is often thought of as tracking fluid intake and monitoring urine color and output to prevent dehydration and overhydration in the senior population, but it is not clear whether these methods would be useful in the older adult population [88]. Contributing to this are changes in body water content percentage, declining renal function, and medications that can alter hydration levels [5]. Additionally, skin turgor can be a simple and noninvasive way to detect possible dehydration.

### 3.2. Clinical and Research Measures

The most utilized standard for assessing hydration in both clinical and research settings is serum osmolality, with a value greater than 295 mOsm/kg serving as a reasonable threshold for dehydration due to water loss, but this cannot diagnose isotonic dehydration and may delay diagnosis in acute clinical situations [49,89]. However, there is no reliable objective method to assess dehydration to both diagnose and confirm the resolution of dehydration [90].

Salivary osmolality can be used as a measure of both hypertonic and isotonic dehydration in older adults, but further research is needed [91], as it can be affected by oral artifacts such as recent fluid consumption and factors influencing saliva flow rate [92]. In a study of older adult subjects (mean age 78), salivary osmolality showed superior diagnostic accuracy when compared with physical signs and urine markers (color and specific gravity) and was able to detect water and solute dehydration, something plasma osmolality cannot detect [93].

Urine osmolality, color, and specific gravity are non-invasive methods of measuring hydration, but urine and plasma osmolality correlate poorly due to differing levels of urea [5]. Other non-invasive clinical measures can be based on changes in body weight, heart rate, sunken eyes, mucosal dryness, skin turgor, and peripheral venous filling [93], but may not be sensitive enough to detect impending or minor dehydration [90].

Bioelectrical Impedance Analysis (BIA) utilizes a mild electrical current to estimate the amount of body water present. This method has not been shown to be useful in assessing hydration in older adults [94]. El Dimassi et al. [95] found that different age groups, ethnic backgrounds, and health conditions may be associated with varying body compositions, which can lead to inaccurate body water estimates. Additionally, the standards from which the BIA measures are related to healthy individuals aged 19–65 with a balanced fluid resistivity, meaning any fluid disorder will result in a biased estimation [95]. Among the issues that can cause a fluid disorder are medications, kidney disease, and heart disease—all of which increase in probability with age.

The Geriatric Dehydration Screening Tool (GDST) has been through several versions, but the newest version is shorter and easier to use, with only four clinical assessments (tongue hydration, axillary hydration and moisture, and body weight) and five questions to assess hydration status in older people [96]. The new version has fair diagnostic accuracy, specifically with those over the age of 75, and can be used as a first step when assessing those who may require osmolality testing [96].

Methods for assessing hydration in research are often like those used in clinical arenas, particularly when clinical data are utilized and can involve BIA, serum and urine osmolality, urine volume, and specific gravity. The new GDST mentioned above is thought to have possible use in both home and research environments [96]. The beverage hydration index (BHI) was developed to be able to compare the effectiveness of various beverages at maintaining hydration compared to (still) water [97], and is widely used in research settings.

Research parameters equate dehydration with urine osmolality greater than 700 mOsm/kg or urine specific gravity greater than 1.020 [20,98]. Additionally, a body weight loss of 3% or more or a 3% gain within 7 days can indicate dehydration [99], and a total body water loss of 2% can indicate the same [89]. Where the older adult population is concerned, a 2015 review found that BIA, urine specific gravity and osmolality, saliva or tear osmolality, tear volume, urine frequency, and urine volume were unreliable diagnostic tools [99]. A summary of various hydration measures is outlined in Table 2.

## 4. What Are Some Strategies to Combat Dehydration?

**Figure 2:**
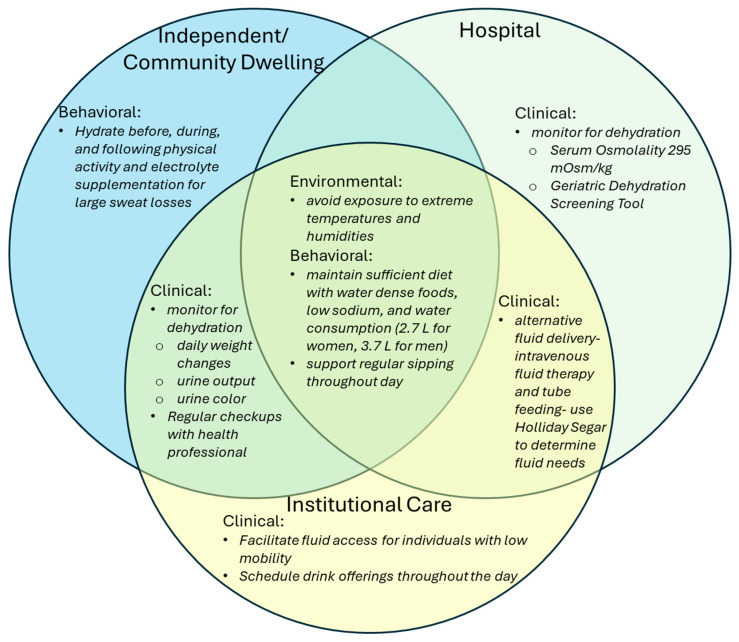
Environmental, behavioral, and clinical interventional strategies to promote hydration for different settings [12,17,23,33,41,93,96,106,107,108,109,110,111,112].

Dehydration is a prevalent yet preventable condition, particularly among older adults, that poses significant health risks if left unaddressed. Effective prevention and management require a holistic strategy encompassing appropriate dietary habits, fluid intake, environmental awareness, physical activity considerations, and individualized medical care [104,105]. Figure 2 outlines different strategies for promoting hydration that have been identified for older adults in different settings. In residential care settings, older adults frequently face hospital admissions for mild to moderate dehydration situations that can lead to serious complications such as delirium, acute confusional states, and hospital-acquired infections [17]. Early identification and intervention are essential, as dehydration is largely reversible when addressed effectively and promptly.

Understanding the unique hydration requirements of older adults is critical, particularly considering varying energy expenditure, environmental conditions, and comorbidities [113]. For instance, factors like high ambient temperatures, physical exertion, or illness can elevate fluid loss, while conditions such as heart or kidney disease may necessitate fluid restriction [114]. Therefore, fluid management must be tailored through regular clinical assessment, considering not only individual health status but also sources of fluid intake, including both beverages and high-water-content foods [17,114].

Ensuring adequate hydration in older adults through personalized strategies enhances nutritional status, supports better clinical outcomes, and improves overall health and quality of life [104]. A multifactorial approach that integrates clinical monitoring, individualized fluid calculation, and lifestyle adjustments remains key to mitigating the adverse effects of dehydration in this vulnerable population.

### 4.1. The Role of Food in Hydration Management

Diet plays a vital role in maintaining proper hydration, particularly for older adults who may have diminished thirst perception or physical limitations affecting fluid intake [17,114]. Consuming foods with high water content such as watermelon, cucumbers, celery, lettuce, zucchini/summer squash, spinach or strawberries, all of which have greater than 90% water content, can significantly contribute to daily fluid needs, making them excellent choices for supporting hydration and benefiting individuals who may struggle to consume adequate fluids throughout the day through nutrition [17,33]. In contrast, high-sodium, salty, or heavily processed foods can exacerbate dehydration by disrupting fluid and electrolyte balance [40,41]. Therefore, maintaining a diet low in sodium and rich in hydrating foods is essential, particularly for vulnerable populations (with the understanding that sodium and other electrolytes are essential to maintaining optimal hydration, with their intake being dictated by the overall needs of the individual).

A holistic and sensory-based approach to mealtimes can improve both fluid and food intake. Presenting foods in a visually appealing way, using herbs and spices to enhance flavor, and offering varied textures and temperatures can stimulate appetite and encourage greater consumption. Providing sufficient time for meals, along with options like soups, smoothies, and hydrating dairy products such as yogurt or cottage cheese, supports both nutritional and hydration needs [17,54].

### 4.2. Fluid Intake

In older adult populations, thirst is triggered only during substantial fluid deficits. As a result, proactive hydration strategies must be prioritized over reactive intake based on thirst cues. Older adults are encouraged to consume fluids steadily throughout the day rather than in large volumes at once, as gastric distension can quickly reduce the sensation of thirst [54]. Studies reveal that older patients on modified diets such as thickened fluids due to swallowing disorders often consume only a fraction of their recommended fluid intake. For individuals with difficulties swallowing or dysphagia, alternatives such as flavored gelatin or smoothies may serve as effective hydrating options [54,115,116].

The Food and Nutrition Board in the Institute of Medicine recommends 3.7 L (~15 cups) total daily water intake (including water in food and beverages) for men aged 19 to >70 years and 2.7 L (~11 cups) per day for women [117]. A person’s age and body composition, as well as comorbidities such as heart failure, kidney disease, and liver dysfunction, which may necessitate fluid restriction, should be taken into consideration when determining individual fluid needs [54]. However, fluid needs vary widely based on environmental temperature, physical activity, and health status in older adults. In cases of fever, for instance, an additional 500 mL of fluid is advised per degree Celsius above 38 °C [63].

Fluid intake does not refer to water alone. Many beverages are able to replace body water similarly to water, including colas, tea, and sports drinks [97], as well as different varieties of milk. Additional ingredients, including carbohydrates, electrolytes, and amino acids, may even facilitate better water absorption. These are discussed in more detail below. Some beverages, such as oral rehydration solutions (ORS) and milk, have been shown to provide better hydration than water alone.

#### 4.2.1. Oral Rehydration Solutions

ORS (which contain sugar, electrolytes, and water) may provide superior benefits over water alone for older adults experiencing dehydration due to an illness. A recent study examined variations within and between different ORS products, including pre-mixed and powdered formulations, as well as osmotic stability over time. The study findings highlight the need to consider both formulation and storage conditions in the development of future ORS products [118]. A recent randomized, placebo-controlled trial investigated advanced ORS formulations enriched with postbiotics, which may help reduce intestinal inflammation during episodes of diarrhea in older adults [119].

#### 4.2.2. Intravenous Fluid Therapy

In critically ill patients, fluid therapy, especially in the form of intravenous solutions, is frequently necessary to maintain circulatory stability and prevent dehydration-related complications [108]. One useful clinical tool for estimating fluid requirements of patients is the Holliday–Segar method, traditionally used for pediatric populations but adaptable for older adults [109,110]. This method calculates daily maintenance fluid needs based on body weight and can be adjusted for older individuals by accounting for age-related physiological changes such as diminished renal function and altered body water composition. Complementary methods like the 4-2-1 rule offer further precision by segmenting fluid intake into hourly needs [120].

Two main categories of intravenous fluids are used for rehydration: crystalloid and colloid solutions. Crystalloids, including normal saline (0.9% sodium chloride), lactated Ringer’s (LR), and glucose-containing solutions (D5W—Dextrose 5% in Water, D10W—Dextrose 10% in Water), are composed of small molecules that pass easily through cell membranes. These are commonly used to restore hydration and electrolyte balance in the extracellular space, though they may lead to tissue edema if used excessively [108,121].

Conversely, colloids consist of larger molecules such as albumin, dextrans, hydroxyethyl starch, and gelatins that remain within the intravascular space and more effectively expand plasma volume. Colloids are often used in scenarios involving severe blood loss or shock, particularly in surgical or intensive care settings. Although they are less likely to cause edema, colloids carry their own risks, including allergic reactions and coagulation issues [108,121,122].

Evidence comparing these solutions is mixed. While some studies report no significant difference in mortality between colloid and crystalloid use, others suggest colloids more effectively reach central venous pressure targets. For example, colloids achieved target central venous pressure in reviewed studies, compared to 79% with crystalloids, and demonstrated more favorable effects on mean arterial pressure in critically ill patients [122].

### 4.3. Environment and Activity

In periods of extreme heat with high humidity, older adults are recommended to remain in air-conditioned environments and to drink plenty of fluids [41]. Individuals are also encouraged to drink plenty of fluids during cold weather when urine excretion is higher and heating areas indoors cause lower humidity, resulting in higher concentrations of water in respiration [23].

The Centers for Disease Control and Prevention (CDC) indicates that regular physical activity is essential for adults aged 65 and older to maintain health, functional ability, and independence [123]. An effective hydration strategy includes drinking fluids before exercise, consistent hydration during physical activity (approximately every 15 to 20 min), and post-activity rehydration [124]. Fluid intake should be tailored to match the duration and intensity of physical exertion, and the inclusion of electrolytes should be considered when necessary to support hydration and electrolyte balance [125]. In cases of prolonged sweating, lasting several hours, consuming sports drinks that contain balanced electrolytes can help replenish sodium that is lost and other essential minerals. When exercise lasts longer than 30 to 60 min, performance can be enhanced by drinking water or carbohydrates, with both independent and additive effects. Many studies support this theory, but the evidence for plain water is less conclusive compared to dilute carbohydrate-electrolyte drinks [126]. However, it is important to moderate the intake of sports beverages, as many are high in added sugars and may contribute to excess caloric consumption [127], leading to weight gain.

## 5. What Are Some Strategies to Improve Hydration?

### 5.1. Regular Fluid Intake Throughout the Day

Healthcare professionals should encourage regular fluid intake in older adults to support optimal physiological functioning. Education should emphasize starting the day with water and maintaining hydration throughout. To promote adequate hydration, consistent fluid intake should be encouraged, including offering water with meals, between meals, and during medication administration [17,107]. Since many older adults limit evening fluid intake to avoid nocturnal incontinence, family caregivers should be educated about hydration strategies. Assessing individual hydration habits is also important, as psychological barriers may influence fluid intake [128]. Reminders, including mobile apps, can help prompt regular drinking, especially in hot or dry environments.

Flavoring water may also encourage individuals to increase fluid consumption. One study, involving 50 patients, assessed fluid intake before and after an intervention that included offering flavored beverages. The postintervention group consistently consumed more fluids than the preintervention group over a week [107].

### 5.2. Fluids Before, During, and After Activity

While many older adults are relatively inactive, others continue to engage in regular and strenuous activity, including exercise. The American College of Sports Medicine, Academy of Nutrition and Dietetics, and Dietitians of Canada have jointly made recommendations on how individuals should hydrate before, during, and after activity [106]. During the 2 to 4 h prior to activity, individuals should consume 2–4 mL of water per pound of body weight. Sipping small amounts of fluid during exercise helps counteract sweat loss, particularly in prolonged or high-intensity workouts. The need for fluid replacement depends on factors such as the length of exercise, intensity, and environmental conditions. In other terms, unless sweating leads to a fluid loss equating to about 2% or more of body weight, research shows minimal performance benefits from drinking during activity.

After physical activity, it is important to rehydrate by replacing both fluids and electrolytes. A general guideline is to drink about 1.5 times the amount of fluid lost during exercise; tracking weight before and after can help estimate this loss. To support this theory, researchers found that consuming fluids in excess of the amount lost, along with appropriate sodium levels, significantly improved fluid retention and helped restore cardiovascular and fluid balance more effectively than water alone [129].

Similar guidance is provided for physically active older adults, although more consideration is recommended, as they may experience blunted thirst, and consuming excessive water and sodium may lead to hyponatremia and hypertension [130]. The International Council on Active Aging generally recommends 8 ounces of water prior to activity, sipping water during, followed by an additional 16–24 ounces of water following. The council recommends using only electrolyte beverages during extreme (“salty”) sweating [127].

### 5.3. Electrolytes

Electrolytes such as sodium, potassium, magnesium, and chloride play a crucial role in maintaining fluid balance and retention in the body. Drinks rich in electrolytes are particularly important during prolonged physical activity or in hot environments where significant sweat loss occurs. Sports drinks are recommended over fruit juices and high-sugar beverages for this purpose [37,131]. Additionally, tablet or powder stick packs containing electrolytes mixed into water have been reported to improve the BHI and noted to be well-tolerated with daily intake [132,133]. Sodium enhances water absorption in the small intestine and helps the body retain fluid [37]. However, aging may alter the effects of electrolytes on increasing fluid retention. Recent studies evaluating hydration characteristics of different beverages in young and old populations have shown that sodium was more effective in maintaining hydration in younger vs. older adults [38,39].

### 5.4. Carbohydrates

Small amounts of carbohydrates, such as glucose, are included in hydration solutions like sports drinks to provide energy, improve water and electrolyte absorption, and add flavor to encourage voluntary drinking [37]. Typical sports drinks contain approximately 6–8% of carbohydrates in solution, including glucose, fructose, sucrose, and maltodextrin that digests quickly, which is ideal for endurance performance and hydration [37,134]. Research indicates sports drinks that utilize a blend of carbohydrates such as glucose and sucrose improve intestinal absorption because different sugars are absorbed through different routes, leading to better replenishment in muscles and enhancing performance [134]. Carbohydrates help maintain blood sugar during long activities and support sodium absorption via glucose-sodium co-transport. Intake of multiple transportable monosaccharides, like glucose and fructose, during prolonged exercise increases gastric emptying, intestinal fluid absorption, and fluid delivery due to different transporters for glucose (SGLT1) and fructose (GLUT 5). Solutions containing both glucose and fructose enhance carbohydrate oxidation and endurance performance compared to single carbohydrate solutions. High intake of glucose plus fructose prevents stomach fullness compared to glucose alone. An optimal glucose to fructose ratio of 1.2:1 to 1:1 maximizes carbohydrate oxidation while minimizing gastrointestinal distress [134].

### 5.5. Amino Acids

Recent research suggests that amino acid- or dipeptide-based beverages can significantly improve fluid balance, as reflected in higher BHI values, which indicate improved fluid retention after consumption [39,135]. This response appears to vary by age, indicating that hydration outcomes may differ across age groups. In addition to fluid balance, amino acid supplements have gained popularity due to the potential to boost anabolic hormone levels, support energy metabolism, enhance mental endurance, and reduce muscle soreness after exercise, in turn aiding in faster recovery—although findings for these outcomes are mixed. These amino acid compounds play a key role in hydration by facilitating the absorption of sodium and water in the small intestine through specialized transporter mechanisms [135]. Additionally, amino acids help maintain fluid homeostasis by supporting intracellular functions that stabilize ion concentrations during physical stress [133,136]. When combined with electrolytes, amino acids offer hydration benefits comparable to traditional carbohydrate-type electrolyte beverages, making them more favorable for individuals who choose to limit carbohydrate intake. Amino acids or dipeptides can serve as a practical alternative to carbohydrates in hydration strategies, while providing broader health benefits [133,135].

### 5.6. Allulose

Allulose is a rare sugar [137] used as a low-calorie sweetener and is found in small traces in fruits like figs and raisins [138]. Allulose is useful in hydration products for those managing calorie or sugar intake and serves as a practical alternative to high-fructose beverages, helping people stay hydrated without the added sugars [133]. A recent study found that allulose has minimal impact on blood glucose and prevents the onset of hyperinsulinemia, hyperglycemia, and insulin resistance associated with a typical Western diet [138].

## 6. What Research Has Been Done on Dehydration?

A large body of research has examined the health effects of dehydration in the general population [14]. Particular attention has also been given to groups at higher risk of dehydration or its more severe consequences, such as children [139,140] and older adults [6,42]. In regard to older adults, hydration research has focused largely on health outcomes and related costs, with a few studies on determining prevalence in certain populations (hospitalized, assisted living) [6,8,10,141]. More research is needed to determine the actual prevalence of dehydration within older adults in institutional settings [142].

Dehydration studies also frequently focus on individuals exposed to greater dehydration risk due to physical activity or hot environments, including athletes [125,143,144] and those in high-risk occupations [145] like military personnel [146] and firefighters [147]. Hot environments are of particular concern with dehydration. The role of hydration on thermoregulation and preventing heat stress/stroke in both athletes [148] and workers [145,149,150] is therefore of interest. Older adults are more susceptible to heat illnesses, and dehydration has been associated with contributing to heat illnesses [32]. However, little research has explored the effects of hydration in older adults under extreme environments and the prevention of heat stress. A small study in old and young men who were thermally dehydrated found that despite experiencing similar levels of sweat loss, the men in the older cohort experienced a higher rectal temperature, drop in plasma volume, and increase in plasma osmolality, were less thirsty and did not perceive they were hotter than those in the younger cohort, and despite drinking similar amounts of water, their physiological markers took longer to normalize [151]. Similarly, a separate study exploring osmoregulation during heat- and exercise-induced dehydration followed by ad libitum rehydration in young and old men found decreased thirst in older men resulting in decreased rehydration [152]. In a dry heat exercise study on pre-pubescent, young, and old males, older males were found to sweat less and have a higher increase in body temperature compared to the younger groups [153].

Changes in cognition and moods due to dehydration are a frequent topic of research. Although methodologies (i.e., measures of dehydration, way of dehydration, degree of dehydration, outcomes) are inconsistent among studies, the body of existing research suggests that dehydration negatively impacts both mood and cognition [154], particularly when extreme or under conditions of heat and/or strenuous activity [154,155]. Some research shows that increasing water consumption can not only boost mood but is also positively associated with shorter simple reaction time, increased attention, and better short-term memory [154]. However, working memory was not improved by increased water consumption, highlighting that not all cognitive processes may benefit. With regard to older adults, two areas of cognitive research have emerged, with one focusing on changes in neurocognition and the other on the relationship between dehydration and dementia [6]. The results of these studies were largely mixed.

Research has found that athletes experience changes in cognition at water losses greater than 2% of body weight and begin to experience detriments to performance at 3–5% losses of body weight [106]. Above that percentage, fluid losses result in changes to sweating, cardiac output, and blood flow. Research on hydration in older athletes is limited. In a 2024 study on the hydration habits of 12 healthy male players of padel (a racket sport popular in Spain), those over the age of 65 were dehydrated following training, and although they consumed the recommended amounts of fluid during the training, they had insufficient intake on average the evening and morning before training [156]. Additionally, those that consumed more than 600 mL per hour experienced less dehydration and increased training intensity. In an earlier study [157], older men and women (54–70 years) drank ad libitum a carbohydrate-electrolyte solution (CES) or distilled water during exercise. The study found that fluid levels were maintained, and the restoration of plasma volume was greater with CES. The study, however, noted that while men preferred the CES, women preferred water.

Optimizing hydration strategies and sports drink composition also is of interest [158]. The BHI was developed to evaluate different beverages’ ability to maintain hydration [97]. Generally, hydration studies have found that older adults excrete less urine following the consumption of the treatment at rest [38,39,159]. These same studies have also found that compositional drink changes alter with age. Older adults may need to consume less salt in order to retain fluids, and amino acids may be an effective substitute for glucose in hydration beverages when glucose addition is not ideal [39,159]. Milk was not as effective at maintaining hydration in an older cohort compared to the young [38].

Dehydration data gathered from longitudinal studies may be useful in preventing chronic diseases and accelerated aging. Using serum sodium as a measure of hydration habits, one study followed 11,255 participants aged 45–66 for 25 years and found that serum sodium above 140 mmol/L was associated with up to 63% increased odds of developing chronic diseases in comparison to a serum sodium level of 138–140 mmol/L [100]. Collecting data from the 2009–2012 National Health and Nutrition Examination Survey (NHANES) of adults aged 51–70, researchers found that underhydration was significantly associated with an increased prevalence of obesity, high waist circumference, insulin resistance, diabetes, low HDL, hypertension, and metabolic syndrome, and after a 3–6 year follow-up, it was determined that underhydration was associated with 4.21 times greater chronic disease mortality [160]. Additionally, a 2020 study aimed to determine what associations could be made between dehydration and long-term cognitive effects. Taking data from the Berlin Aging Study II (BASE-II), a longitudinal association was found between dehydration and declining cognitive function [161]. More longitudinal studies would add to the preventative aspects of proper hydration.

### 6.1. Limitations

Studies on hydration in older adults are mostly conducted in hospitalized patients and residents in care homes. Research focusing on older individuals who live independently or in a family unit should be explored more. Studies used a variety of metrics in determining dehydration, including some that may not be relevant in older populations, such as urine color. Studies should include serum osmolality as a hydration marker to allow for better comparisons between studies. Comorbidities may greatly affect hydration status and need to be carefully considered in the study design and data analysis. More work should be done to determine how hydration strategies may be altered to maintain hydration, giving special attention to personal preferences for beverage intake in order to maximize fluid volume.

### 6.2. Future Research Directions

With an ever-increasing interest in novel nutritional approaches to improving health, future studies should focus on the development and use of functional nutritional beverages to promote hydration in older adults. These may need to be explored with regard to common comorbidities such as heart disease, diabetes, and dementia. Hydration strategies for physically active older adults should also be probed, as many continue to be engaged in programs of regular exercise. Longitudinal studies that follow adults from middle to old age may better elucidate how hydration status may alter quality of life and lifespan.

## 7. Conclusions

Hydration is beneficial in all seasons of life. Aging alters our ability to regulate and maintain body water. As we age, our total body water decreases, our thirst decreases, resulting in less desire to rehydrate, our absorption of water and nutrients changes, and our ability to regulate levels of water and sodium decreases as urine becomes more dilute. Dehydration in aging can greatly affect the quality of life, resulting in changes to cognitive and physical functions, leading to ill-health or disease. Despite this knowledge, dehydration continues to be prevalent among older adults. Promoting lifelong healthy hydration habits should begin with early education on effective hydration strategies and the recognition of dehydration symptoms. Individuals should be encouraged to regularly self-monitor their hydration status using simple at-home methods—such as tracking changes in body weight (especially after physical activity), monitoring urine output, and observing urine color. In addition, annual clinical checkups should include hydration biomarker assessments. Together, these practices can help reduce long-term health risks and support a higher quality of life throughout the aging process.

While many recommendations have been made on how to encourage better hydration practices for older adults living in different home environments, with varying degrees of support, a standard methodology is needed to better track hydration and to evaluate different interventions more consistently. Additional research is needed to understand how different comorbidities alter hydration needs and what dietary changes and novel beverage delivery systems may improve hydration in this vulnerable population.

## Figures and Tables

**Table 1:** Diseases that contribute to dehydration status.

Comorbidity	Pathway to Dehydration	Study
Acute coronary syndrome	Hyperosmolality leads to increased risk of renal injury, cerebral ischemic events, and increased cardiovascular mortality	[5,64,65]
Arterial/Venous thrombosis	Increased blood viscosity and hematocrit	[5,11,66]
Cardiovascular disease	Fluid buildup leading to fluid restriction	[24,67,68]
Dementia	Impaired memory	[21,69,70]
	Lack of proper care or support	
	Impaired ability to detect hunger and thirst	
	Dysphagia	
Diabetes	Increased thirst	[29,71,72]
	Increased urine output	
Dysphagia	Difficulty swallowing	[42,73,74]
Incontinence	Fluid restriction to control frequency	[75,76,77]
Influenza	Diarrhea	[63,78,79]
	Vomiting	
	Fever	
	Increased sweating	
Kidney disease	Increased fluid loss due to urine output	[80,81,82]

**Table 2:** Measures of hydration.

Measure	Description	Source
Bioelectrical Impedance Analysis (BIA)	Estimates body water Not accurate in disease population	[90,94,99]
Blood Properties		
BUN/Creatinine Ratio	>20 non-specific, but may indicate isotonic dehydration	[93]
Plasma Copeptin	>6.79 pmol/L or >10.6 pmol/L for men and >6.5 pmol/L for women	[19,20]
Plasma Osmolality	>300 mOsm/kg	[42]
Serum Osmolality	>295 mOsm/kg, not accurate for isotonic	[5,10,42,49,89]
Serum Sodium	>142 or >144 mmol/L	[19,100]
Cardiovascular		
Heart Rate	>90–100 beats per minute, not sensitive in older adults	[5,93]
Systolic Blood Pressure	<100 mmHg	[5,93]
Peripheral Venous Filling	Non-invasive clinical indicator	[93]
Geriatric Dehydration Screening Tool (GDST)	Utilizes a combination of clinical examination and hydration questions for patient	[96]
Eyes	Non-invasive clinical indicator Appear sunken	[93]
Mass		
Body Water loss	≥2%	[20,89]
Total Body Mass	≥3%	[99]
Mucosal Dryness	Non-invasive clinical indicator	[93]
Saliva		
Osmolality	>93 mOsm/kg or	[91,92,93,101]
	>94–97 mOsm/kg in Emergency Department	
Flowrate	Poor indicator	[93]
Skin Turgor	Non-invasive clinical indicator	[93]
Tears		
Osmolality	Not accurate measure, may require waiting a period with eyelids closed	[99,102]
Volume	Not accurate measure	[99]
Thirst	Poor indicator in older adults	[8,12,50]
Tracers	Includes D_2_O, used mainly in research to determine hydration rates	[5,42,45]
Urine		
Color	(dark yellow), >6 on 8-point scale, simplistic, but unreliable	[20,49]
Frequency	Not accurate	[99]
Specific Gravity	1.020	[20,98]
Osmolality	700 mOsm/kg	[20,98]
Volume	0.5 L/24 h	[20,103]

## Abbreviations

The following abbreviations are used in this manuscript:

ACE inhibitors	Angiotensin-converting enzyme inhibitors
BASE-II	Berlin Aging Study II
BHI	Beverage Hydration Index
BIA	Bioelectrical impedance analysis
BUN	Blood Urea Nitrogen
CDC	Center for Disease Control
C	Celsius
CES	carbohydrate-electrolyte solution
Cr	Creatinine
d/t	due to
D5W	Dextrose 5% in Water
D10W	Dextrose 10% in Water
F	Fahrenheit
g	grams
GDST	Geriatric Dehydration Screening Tool
GFR	Glomerular Filtration Rate
GLUT 5	Glucose transporter 5
LR	Lactated Ringers
L	liters
mL	milliliters
mmol/L	millimoles per liter
mOsm/kg	Milliosmoles per kilogram
NHANES	National Health and Nutrition Examination Survey
NSAIDs	Nonsteroidal anti-inflammatory drugs
ONS	Oral Nutrition Supplements
ORS	Oral Rehydration Solutions
pmol/L	picomoles per liter
POMS	Profile of Mood States
SGLT1	Sodium/glucose cotransporter 1
SLIM	Satiety Labeled Intensity Magnitude
WHO	World Health Organization
